# Epidemiological Characteristics and Spatio-Temporal Distribution of Hepatitis A in Spain in the Context of the 2016/2017 European Outbreak

**DOI:** 10.3390/ijerph192416775

**Published:** 2022-12-14

**Authors:** María Guerrero-Vadillo, Marina Peñuelas, Ángela Domínguez, Pere Godoy, Diana Gómez-Barroso, Nuria Soldevila, Conchita Izquierdo, Ana Martínez, Nuria Torner, Ana Avellón, Cristina Rius, Carmen Varela

**Affiliations:** 1 Doctorate Programme in Biomedical Sciences and Public Health, National University of Distance Education (UNED), 28015 Madrid, Spain; 2 National Centre for Epidemiology, Instituto de Salud Carlos III, 28029 Madrid, Spain; 3 Departament de Medicina, Universitat de Barcelona (UB), 08036 Barcelona, Spain; 4 Consortium for Biomedical Research in Epidemiology and Public Health (CIBERESP), 28029 Madrid, Spain; 5 Department of Medicine, Institut de Recerca Biomédica de Lleida (IRBLLeida)-Universidad de Lleida, 25008 Lleida, Spain; 6 Agència de Salut Pública de Catalunya, 08005 Barcelona, Spain; 7 Hepatitis Unit, National Centre of Microbiology, Instituto de Salud Carlos III, 28222 Majadahonda, Spain; 8 Agència de Salut Pública de Barcelona, 08023 Barcelona, Spain

**Keywords:** hepatitis A virus, epidemiological surveillance, MSM, outbreaks, space-time clustering, urban population

## Abstract

The aim of our study was to describe the results of the epidemiological surveillance of hepatitis A infections in Spain in the context of the 2016/2017 European outbreak, particularly of hepatitis A outbreaks reported in the MSM population, incorporating the results of a spatio-temporal analysis of cases. Hepatitis A cases and outbreaks reported in 2016–2017 to the National Epidemiological Surveillance Network were reviewed: outbreaks in which some of the cases belonged to the MSM group were described, and clusters of hepatitis A cases in men and women were analysed using a space–time scan statistic. Twenty-six outbreaks were identified, with a median size of two cases per outbreak, with most of the outbreak-related cases belonging to the 15–44 years-old group. Nearly 85% occurred in a household setting, and in all outbreaks, the mode of transmission was direct person-to-person contact. Regarding space–time analysis, twenty statistically significant clusters were identified in the male population and eight in the female population; clusters in men presented a higher number of observed cases and affected municipalities, as well as a higher percentage of municipalities classified as large urban areas. The elevated number of cases detected in clusters of men indicates that the number of MSM-related outbreaks may be higher than reported, showing that spatio-temporal analysis is a complementary, useful tool which may improve the detection of outbreaks in settings where epidemiological investigation may be more challenging.

## 1. Introduction

Hepatitis A virus is a picornavirus of the genus *Hepavirus*, with only one serotype distributed worldwide [1]. It is transmitted mainly from person to person by the fecal-oral route and by ingestion of contaminated water or food. The virus typically spreads among close contacts, such as household members, with children being the group with the greatest rates of infection [2].

The disease presents different patterns of endemicity worldwide, being heavily linked to poor hygiene and sanitary conditions and other parameters of low level of development. Western Europe, like other high-income regions such as the United States, Canada or Japan, is considered an area of very low/low endemicity of hepatitis A, although infections in high-risk groups and foodborne outbreaks are still being notified [3]. In 2016, around 12,500 cases were reported in the EU/EEA (2.4 cases per 100,000 population), with the highest notification rate in the 5–14 years-old group (7.9 confirmed cases per 100,000 population). In all age groups, males were more affected than women [4].

The disease may be prevented by vaccination. In Spain, inactivated vaccines are available, which are safe and highly immunogenic, and confer long-term protection against the infection [5,6]. As a low-endemicity country, vaccination is currently recommended in high-risk groups, such as groups with HIV infection, alcoholism and chronic liver disease, as well as men who have sex with men [7], although in certain regions (Catalonia, Ceuta and Melilla) the vaccine is also included in the childhood immunization schedule.

Despite the low endemicity of hepatitis A in Spain, important outbreaks still occur, especially in certain groups such as MSM [8]. In this specific population, several risk factors, such as oral-anal sex and drug consumption, have been described [9,10]. During 2016/2017, a multi-country outbreak of hepatitis A affecting principally MSM took place in the EU/EEA, with around 4000 cases; 1400 cases were confirmed, and 54% of them were hospitalised. Molecular analysis revealed that three different strains belonging to genotype IA (VRD_521_2016, RIVM-HAV16-090 and V16-25801) were involved [11].

The aim of our study was to describe the results of the epidemiological surveillance of hepatitis A infections in Spain in the context of the 2016/2017 European outbreak, particularly of hepatitis A outbreaks reported among the MSM population, incorporating the results of a spatio-temporal analysis of cases.

## 2. Materials and Methods

### 2.1. Study Area

**Figure 1:**
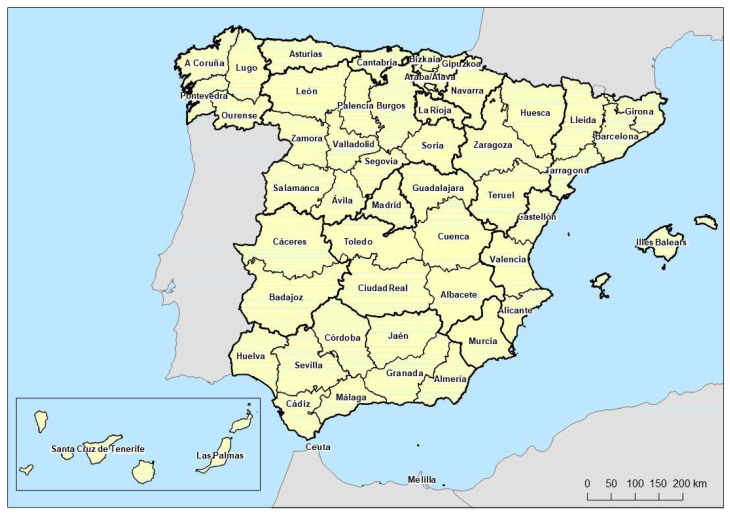
Political map of Spain.

Spain is divided into 17 autonomous regions (further subdivided into 50 provinces and 2 autonomous cities, with the 8131 municipalities being the smallest administrative units) (Figure 1). According to the Atlas Estadístico de las Áreas Urbanas en España 2021 of the Ministry of Transport, Mobility and Urban Agenda (MITMA in Spanish), the population distribution is not homogeneous, since 69% of the Spanish population live in large urban areas with more than 50,000 inhabitants; in fact, 76% of the employment of the country is concentrated in these areas. Conversely, 86.7% municipalities are considered as non-urban, which represents 79.3% of the area of the country, although only 17.3% of Spanish inhabitants live there. According to this methodology, Spanish territory could be divided into three types of areas based on their relation with urban phenomenon, classifying the municipalities into large urban areas (which can be composed of one or more municipalities, and whose denomination is determined by the main city or cities), small urban areas (which are subdivided into 2 categories: municipalities of 20,000–50,000 or 5000–20,000 inhabitants, which do not belong to large urban areas) and non-urban [12].

### 2.2. Data Sources

In Spain, hepatitis A has been a notifiable disease since 1995 [13]; therefore, both probable and confirmed cases (a person with an epidemiological link who meets clinical criteria and a laboratory-confirmed case, respectively) and outbreaks (two or more cases with an epidemiological link) must be reported by regional public health authorities to the National Epidemiological Surveillance Network (RENAVE in Spanish), which is managed by the National Centre of Epidemiology [14].

### 2.3. Hepatitis A Outbreaks

Outbreaks reported in 2016 and 2017 to RENAVE were reviewed, selecting those in which some of the cases belonged to the MSM group according to the information registered in a free text field for comments. Variables analysed included case occurrence year, age groups (0–14, 15–44, 45–64 and >64 years old) and sex of cases, duration of outbreaks (difference in days between the onset of symptoms of the first and last case of the outbreak), size (number of cases per outbreak), setting, mode of transmission, control measures (health education, contact tracing and close contacts vaccination), molecular information about the virus isolated (genotype, subgenotype and strains) and municipality.

### 2.4. Hepatitis A Cases

Cases reported to RENAVE were also collected. The date of onset of symptoms was used, or the nearest one if it was not known (date of diagnosis, date of hospitalization, etc.). The municipality was assigned as the one where the transmission occurred, or the municipality of residence if this information was not available.

#### 2.4.1. Temporal Trends

Annual cumulative incidences per 100,000 inhabitants were calculated, globally and for sex and age groups (0–14, 15–29, 30–49, 50–64 and ≥65 years old), using as denominator the Spanish population on 1st January of each year obtained from the National Institute of Statistics (INE in Spanish) [15]. Male-to-female ratio was also calculated dividing the cumulative incidence (CI) of men by the CI of women. The 2016–2017 period was compared with previous (2012–2015) and following years (2018 and 2019).

#### 2.4.2. Space–Time Cluster Analysis

In line with the results obtained in the descriptive analysis of cumulative incidences, hepatitis A cases aged 15–49 reported in 2016 and 2017 were selected and divided into two groups according to their sex (men and women). Both groups were independently analysed at the municipal level using the space–time scan statistic developed by Kulldorff et al. [16], assuming a Poisson distribution. The statistic detects and evaluates the presence of space–time aggregations of cases. A scan of the study area was performed using a cylindrical window which moves across the centroids of the municipalities; it is constituted by a circular base which defines the spatial dimension of each cluster (and varies from a 0 to 25 km radius) and a height which defines the temporal dimension (and varies from 0 to 80 days, corresponding with two average incubation periods). The cylindrical window moves through space and time, the null hypothesis being that the risk inside and outside the window is the same. For each window, a likelihood ratio test was performed: the cluster with the highest likelihood is the least likely to have occurred by chance. Furthermore, a *p*-value was assigned to each cluster using a Monte Carlo test, by which 999 random simulations were performed. For the analyses, the 2017 populations with corresponding age groups and sexes obtained from INE were used. Additional analyses were performed in both the male and female case groups, considering a maximum temporal cluster duration of 40 and 120 days.

Municipalities were classified according to the Atlas Estadístico de las Áreas Urbanas of MITMA (large urban areas, small urban areas and non-urban) [12], as described above.

### 2.5. Statistical Analyses

Categorical variables were presented as total number and percentage, and continuous variables as median and interquartile range (IQR; p25–p75). Differences in medians were analysed using Mann–Whitney’s test. The association between annual cumulative incidences and male-to-female ratio was measured by a Pearson correlation test. Differences between the percentage of municipalities classified as large urban areas in male and female clusters were evaluated using a chi-square test. Statistical significance was defined as *p*-value < 0.05.

Analysis was performed using Stata 16, and SaTScan 9.7 was used for the space–time analysis. Maps were generated using QGIS 3.16.

## 3. Results

### 3.1. Outbreaks Related to MSM

Only 26 (8.9%) out of 293 outbreaks reported in 2016 and 2017 mentioned transmission among MSM; 24 of them (92.31%) took place in 2017. Outbreak characteristics are shown in Table 1. The median duration of outbreaks was 28 days (IQR: 18–34 days), but considerable differences were present depending on the year. The median size of outbreaks was two cases, without differences according to year of notification.

Most outbreaks (84.62%) occurred in a household setting. These outbreaks presented significantly smaller size than outbreaks in other settings (two vs. three cases, *p*-value: 0.037); they also presented shorter duration (26.5 vs. 54 days, *p*-value: 0.128). In all outbreaks, the mode of transmission was direct person-to-person contact. Information regarding control measures was available in 14 outbreaks, health education being mentioned in 8 outbreaks, contact tracing in 8 outbreaks, and close contacts vaccination (applied or at least recommended) in 10 outbreaks. None of the first cases of each outbreak were vaccinated (information available in only 5 outbreaks).

The outbreaks included a total of 141 cases, without information about sex and/or age group in 21 cases out of 10 outbreaks. Of 120 cases with information in both variables, 118 (98.33%) were men, and 106 (88.33%) belonged to the 15–44 years-old group (Table 1).

In only five outbreaks from three different autonomous regions, microbiological information about hepatitis A virus was available. Genotype IA was identified in all of them; the strains detected were VRD_521_2016 in four outbreaks and the three strains related to the epidemic in MSM (VRD_521_2016, RIVM-HAV16-090 and V16-25801) in one outbreak.

**Figure 2:**
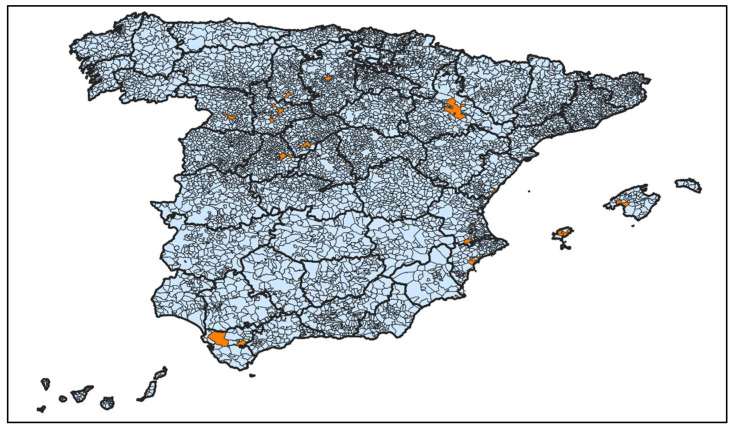
Spatial distribution of MSM-related outbreaks, municipal level.

The outbreaks were reported in eight autonomous regions in the centre, east and south of the country. Information about municipality was available in 24 outbreaks (Figure 2).

### 3.2. Temporal Trends and Male-to-Female Ratio

**Figure 3:**
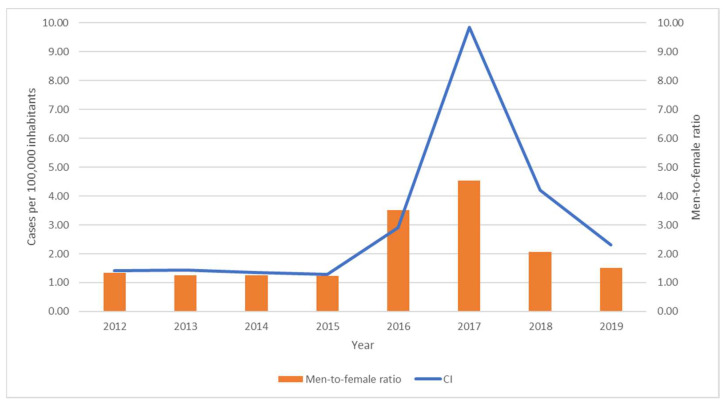
Cumulative incidence (CI) and male-to-female ratio, 2012–2019.

During the 2012–2018 period, 11,534 cases were notified. From 2012 to 2015, cumulative incidence remained stable around 1.37 cases per 100,000 inhabitants, rising later during 2016–2017 (reaching a maximum of 9.85 cases per 100,000 inhabitants in 2017), followed by a decrease in 2018 and 2019. A similar trend was observed regarding the male-to-female ratio, which achieved 3.51 and 4.53 in 2016 and 2017, respectively (Figure 3), the Pearson correlation being between this parameter and the cumulative incidence of 0.874.

**Figure 4:**
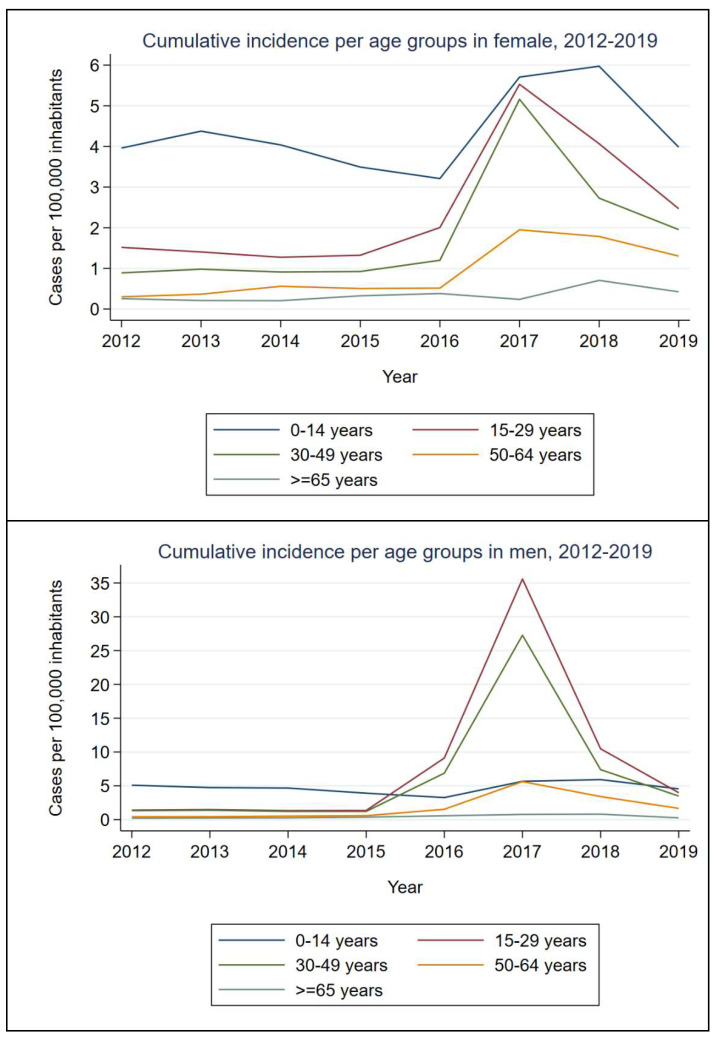
Cumulative incidence by sex and age groups, 2012–2019.

Temporal trends were also studied by sex and age groups. In women, an inversely proportional association was observed between cumulative incidence and age, with the 0–14 years-old group having the greatest one during the entire period, with 5.97 cases per 100,000 inhabitants in 2018. Nevertheless, in addition to the youngest population, a considerable increase of cases was observed in women aged 15–64 in 2017 and 2018. Regarding men, the inversely proportional association previously described was observed until 2015, with 3.92 cases per 100,000 inhabitants in the 0–14 years-old group that year. Since then, the cumulative incidence has risen drastically in individuals aged 15–29 and 30–49, which became the population with the highest number of cases from 2016 to 2018 (maximum of 35.58 and 26.27 cases per 100,000 inhabitants in 2017, respectively); in 2019, the 0–14 years-old group was again the most affected one. Cumulative incidence in the population older than 64 years in both men and women remained less than one case per 100,000 inhabitants during the entire period (Figure 4).

### 3.3. Space–Time Cluster Analysis

During the 2016–2017 period, 4766 cases in men and women aged 15–49 were reported; information about the municipality was available in 99.24% of them.

**Figure 5:**
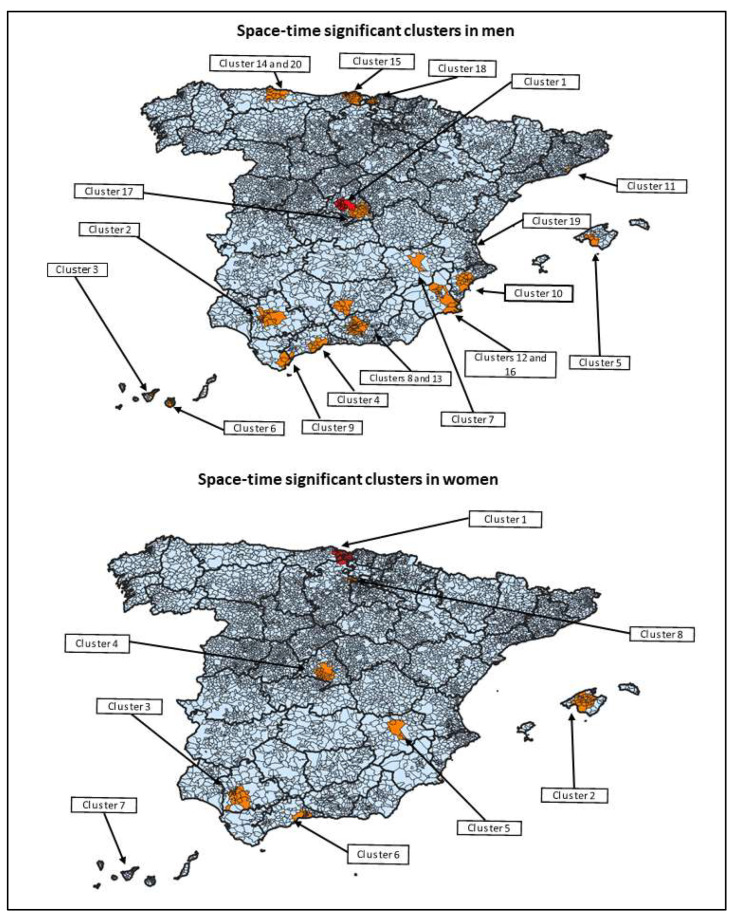
Map of municipalities involved in the space–time significant clusters of hepatitis A.

Considering a maximum temporal cluster size of 80 days, 20 statistically significant clusters were identified in men (Figure 5, Table 2). The most likely cluster was situated in a big town at the centre of the country and 16 neighbouring municipalities; 14/17 municipalities were classified as large urban areas. The cluster consisted of 257 observed cases from 1 February 2017 to 21 April 2017, with 36.98 being the number of expected cases. The other 19 clusters were also detected in large urban areas, such as cluster two, cluster three, cluster four, cluster five or cluster eleven. Most clusters were detected from January to July 2017. Cluster ten coincided with an outbreak of two people reported in the same municipality; no other coincidences in space and time among clusters in men and outbreaks with person-to-person transmission involving the MSM population were detected.

In the female population, eight statistically significant clusters were detected (Figure 5, Table 2). The most likely cluster was located in an area composed of 31 municipalities of three different autonomous regions in the north of the country; 7/31 municipalities belonged to the same large urban Area, whereas 22/31 municipalities were classified as non-urban. The cluster was constituted by 13 observed cases from 2 June 2017 to 23 June 2017, with 0.10 expected cases. Similar to the male population, most clusters were also detected in other large urban areas, such as cluster two, cluster three, cluster four, cluster six and cluster seven; cluster eight included only municipalities classified as non-urban or small urban areas (eight and one municipalities, respectively). All clusters were detected throughout 2017.

Clusters in men presented more total observed cases and number of affected municipalities than clusters in women (937 vs. 121 observed cases, and 312 vs. 159 municipalities, respectively). Clusters in men included a greater percentage of municipalities classified as large urban areas than clusters in women, although these differences were not statistically significant (50.0% vs. 43.4%, *p*-value: 0.175). It was observed that seven out of eight clusters in women were detected in the same areas as clusters in men (sharing at least one municipality); clusters in men started before those in women on several occasions.

Analyses performed using a maximum temporal cluster size of 40 and 120 days obtained similar results, except by the percentage of municipalities classified as large urban areas, which was greater in female than in male clusters in the 120-day analysis (46.15% vs. 44.4%, *p*-value: 0.713) (see Supplementary Material).

## 4. Discussion

Outbreaks in the MSM population accounted for less than 10% of total hepatitis A outbreaks reported during the 2016/2017 period. In those outbreaks, transmission occurred mainly in small groups of cohabitants or partners, since the household was the main setting and the outbreaks presented a small duration and size, with a median of 28 days and two cases, respectively. Most cases were men from ages 15 to 44. In the five outbreaks with genotyping information available, at least the VRD_521_2016 strain, which was also the most detected in other countries, was identified [17,18].

Like in other regions of Europe, the incidence of this disease peaked in 2017, decreasing gradually during 2018 and 2019 [11]. This tendency was quite correlated with the male-to-female ratio, which also peaked in 2017. This parameter was previously used in different studies including hepatitis A [19], shigellosis [20] and sexually transmitted infections [21] as a proxy for MSM transmission. Analyzing the incidence by sex and age groups, a similar pattern in both sexes was observed until 2015, with the individuals younger than 14 years old presenting the highest incidence. Nevertheless, in the 2016–2018 period, men aged 15–29 years became the group with the highest incidence, followed by the 30–49 years-old group. This distribution of cases reflects the epidemiological situation of that moment, similar to that reported in other countries during the European outbreak [18,22].

Even though immunization against hepatitis A is highly recommended in MSM, several studies suggest that the vaccination coverage in this population group in Spain is lower than expected [9,10], which has also been observed in other European countries [23,24]. Regan et al. estimated that a proportion of immune individuals greater than 70% in the MSM population is necessary to prevent major outbreaks [25]. The high proportion of non-immunized individuals, along with high-risk sexual behaviours involving highly interconnected groups, were the main triggers for the European outbreak [11], which was initiated following the EuroPride festival celebrated in Amsterdam [26]. The decline in the cumulative incidence throughout 2017 and 2018 occurred due to several reasons: a decrease in the proportion of susceptible individuals in groups at higher risk of infection; a strengthening in the promotion of vaccination among MSM, along with a prioritization of vaccination in this specific group (especially important in the context of the vaccine shortage in 2017); and numerous health education campaigns launched through websites and apps [27,28].

The space–time analysis reveals differences in the distribution of cases in men and women. The number of clusters detected in men was larger, with more observed cases and involved municipalities. Differences in the duration of clusters were also present: most clusters detected in men had a duration longer than 70 days, whereas only one cluster in women showed this characteristic. In men, an excess of cases seems to be present in at least the entire first semester of 2017 in certain areas, which could indicate a maintained transmission of the disease during this period in this specific population group; whereas in women, the aggregation of cases is more limited in terms of number of observed cases, duration and number of municipalities, which suggests that these cases are involved in outbreaks restricted in time and space. These differences in the clustering of cases between men and women have also been described in other studies. Lanini et. al, in an analysis of hepatitis A cases reported in the Italian region of Lazio between January 2016 and March 2017, observed a disparate temporal distribution of hepatitis A clusters between men, women and children, with the highest number of cases presented in the male cluster [29]. Detection of clusters in municipalities classified as large urban areas was frequent in both sexes; nevertheless, the percentage of municipalities with this classification was higher in men than in women, although without statistically significant differences. Moreover, clusters of both men and women were observed in the same regions of the country.

No correlation was observed between outbreaks related to MSM and clusters in men, which may be due to several factors: an underreporting of outbreaks; a lack of information regarding the mode of transmission and sexual practices/orientation that impedes their identification in the outbreak database; and the difficulties that may arise in the investigation of certain outbreaks, in which it may be complex to identify the epidemiological link between cases. Nevertheless, both outbreaks related to MSM and clusters in men were detected mainly in 2017.

Spatio-temporal scan statistics have previously been used in the epidemiological analysis of communicable diseases [30,31,32], particularly assessing the usefulness of these tools for an earlier detection of outbreaks that would improve their management and control. Kummerer et al. assessed the effectiveness of SatScan in the detection of tuberculosis outbreaks. They showed that using this tool, the local health department could recognize the outbreaks several months in advance [33]. Stelling et al. evaluated the performance of both WHONET and SatScan in the identification of *Shigella* spp. outbreaks, detecting a concordance between several identified clusters and outbreaks reported to public health authorities, in addition to other aggregations of cases that could be previously unidentified events [34].

Several limitations have been identified in the present study. Firstly, information related to sexual orientation or sexual practices is not systematically collected, which may lead to an underreporting of outbreaks with MSM transmission during the study period, and may explain the weak coincidence detected between male clusters and outbreaks; furthermore, age or sex was missing for several outbreak-related cases, and control measures were only reported in 54% of outbreaks. Most outbreaks were detected in a household setting, where the epidemiological link was easier to identify than in other settings, which may be underrepresented in the outbreak investigation. Information about the genotype and the strain of the virus isolated was only available in five outbreaks (19%), which hampers the interpretation of the results. Secondly, municipality of residence was assigned in case of missing information regarding where the transmission occurred, which may be a confounding factor in the space–time analyses. Thirdly, differences between autonomous regions regarding the outbreak investigation and the reporting of hepatitis A cases and outbreaks to RENAVE may affect the results. Finally, as the hepatitis A outbreak affecting Europe, began in mid-2016 and the number of cases have increased since then, the progressive establishment of epidemiological surveillance could have led to increased case reporting during 2017 compared to the previous year.

## 5. Conclusions

We described the epidemiological situation of hepatitis A in Spain in 2016/2017, when a European outbreak related to person-to-person transmission involving the MSM population was ongoing. Most of the MSM-related outbreaks occurred in the household setting. The elevated number of cases detected in male clusters indicates that the number of MSM-related outbreaks may be higher than reported, which shows that spatio-temporal analysis is a complementary, useful tool in the epidemiological surveillance of infectious diseases, which may improve the detection of outbreaks in settings where epidemiological investigation may be more challenging due to the difficulty of identifying links between cases. In addition, genome sequencing may also contribute to the identification of outbreaks belonging to multi-country public health events. Initiatives aimed at increasing hepatitis A vaccination coverage in specific risk groups may improve the prevention and control of these outbreaks.

## Figures and Tables

**Table 1:** Epidemiological characteristics of hepatitis A outbreaks among MSM and related cases, 2016–2017.

OUTBREAKS AMONG MSM				
Variables		Total	2016	2017
Total outbreaks, *n* (%)		26	2 (7.69%)	24 (92.31%)
Duration of outbreak in days, median (IQR *)		28 (18–34)	36 (18–54)	28 (16–34)
Cases per outbreak, median (p25–p75)		2 (2–2)	2 (2–2)	2 (2–2)
Settings, *n* (%)	Household	22 (84.62%)	1 (50.00%)	21 (87.50%)
	Other	4 (15.38%)	1 (50.00%)	3 (12.50%)
**CASES IN OUTBREAKS AMONG MSM ^**				
**Variables**		**Total**	**2016**	**2017**
Total number of cases		120	2	118
Male sex, *n* (%)		118 (98.33%)	2 (100%)	116 (98.31%)
Age groups, *n* (%)	0–14 years	1 (0.83%)	0	1 (0.85%)
	15–44 years	106 (88.33%)	2 (100%)	104 (88.14%)
	45–64 years	13 (10.83%)	0	13 (11.02%)
	≥65 years	0	0	0

* IQR: interquartile range; ^ Cases with information of sex and age groups.

**Table 2:** Space–time significant clusters of hepatitis A in 15–49 years-old men and women, Spain, 2016/2017.

Cluster	Start Date	End Date	Duration (Days)	Observed	Expected	Relative Risk	*p*-Value	Total Number of Municipalities (N Classified as Large Urban Area)
STATISTICALLY SIGNIFICANT SPACE-TIME CLUSTERS IN MEN								
1	1 February 2017	21 April 2017	79	257	36.98	7.36	<0.001	17 (14)
2	31 December 2016	15 March 2017	74	113	12.12	9.57	<0.001	31 (20)
3	1 May 2017	17 July 2017	77	69	5.88	11.92	<0.001	16 (9)
4	20 February 2017	10 May 2017	79	80	9.90	8.22	<0.001	13 (8)
5	6 December 2017	6 December 2017	0	12	0.06	187.12	<0.001	8 (6)
6	5 May 2017	17 July 2017	73	41	6.96	5.94	<0.001	13 (8)
7	7 March 2017	16 March 2017	9	12	0.21	56.39	<0.001	1 (1)
8	1 August 2016	13 October 2016	73	22	1.71	12.96	<0.001	12 (4)
9	24 March 2017	5 June 2017	73	25	2.49	10.08	<0.001	9 (4)
10	29 March 2017	14 June 2017	77	42	8.50	4.99	<0.001	19 (8)
11	21 April 2017	6 July 2017	76	55	14.72	3.77	<0.001	1 (1)
12	28 April 2017	16 July 2017	79	40	8.04	5.01	<0.001	8 (3)
13	16 February 2017	4 May 2017	77	31	5.14	6.06	<0.001	47 (27)
14	15 February 2017	3 May 2017	77	28	4.28	6.58	<0.001	13 (9)
15	8 January 2017	28 March 2017	79	23	2.78	8.32	<0.001	35 (3)
16	17 April 2017	25 April 2017	8	8	0.12	69.24	<0.001	10 (2)
17	21 March 2017	2 June 2017	73	38	9.84	3.89	<0.001	35 (13)
18	20 April 2017	6 July 2017	77	22	3.36	6.57	0.001	20 (12)
19	4 October 2017	4 October 2017	0	3	0.00	2791.78	0.004	1 (1)
20	7 June 2017	23 August 2017	77	16	1.87	8.59	0.007	3 (3)
**STATISTICALLY SIGNIFICANT SPACE-TIME CLUSTERS IN WOMEN**								
1	2 June 2017	23 June 2017	21	13	0.10	132.21	<0.001	31 (7)
2	6 December 2017	20 December 2017	14	13	0.21	63.78	<0.001	33 (6)
3	24 January 2017	10 March 2017	45	20	1.36	15.13	<0.001	28 (24)
4	13 April 2017	7 June 2017	55	34	5.83	6.07	<0.001	27 (21)
5	3 March 2017	21 March 2017	18	8	0.07	110.72	<0.001	4 (1)
6	15 March 2017	22 May 2017	68	15	1.05	14.61	<0.001	15 (4)
7	16 June 2017	1 September 2017	77	13	0.86	15.33	<0.001	12 (6)
8	22 November 2017	27 December 2017	35	5	0.02	205.91	0.0013	9 (0)

## Data Availability

The datasets generated and analysed during the current study are available from the corresponding author on reasonable request.

## Supplementary Materials

The following supporting information can be downloaded at: https://www.mdpi.com/article/10.3390/ijerph192416775/s1, Figure S1: Map of municipalities involved in the space–time significant clusters of hepatitis A in 15–49 years-old men and women, Spain, 2016/2017, maximum temporal cluster size of 40 days; Table S1: Space–time significant clusters of hepatitis A in 15–49 years-old men and women, Spain, 2016/2017, maximum temporal cluster size of 40 days; Figure S2: Map of municipalities involved in the space–time significant clusters of hepatitis A in 15–49 years-old men and women, Spain, 2016/2017, maximum temporal cluster size of 120 days; Table S2: Space–time significant clusters of hepatitis A in 15–49 years-old men and women, Spain, 2016/2017, maximum temporal cluster size of 120 days.
