# Supplementary material for: Epidemiological Characteristics and Spatio-Temporal Distribution of Hepatitis A in Spain in the Context of the 2016/2017 European Outbreak
Source: Int J Environ Res Public Health. 2022 Dec 14;19(24):16775. doi: 10.3390/ijerph192416775 (PMC9778781; doi:10.3390/ijerph192416775)
Supplement: Supplementary file 1 [file ijerph-19-16775-s001.zip › ijerph-2052702-supplementary.pdf]

Supplementary Figure S1. Map of municipalities involved in the space-time significant clusters of hepatitis A in 15-49 years-old men and women, Spain, 2016/2017, maximum temporal cluster size of 40 days

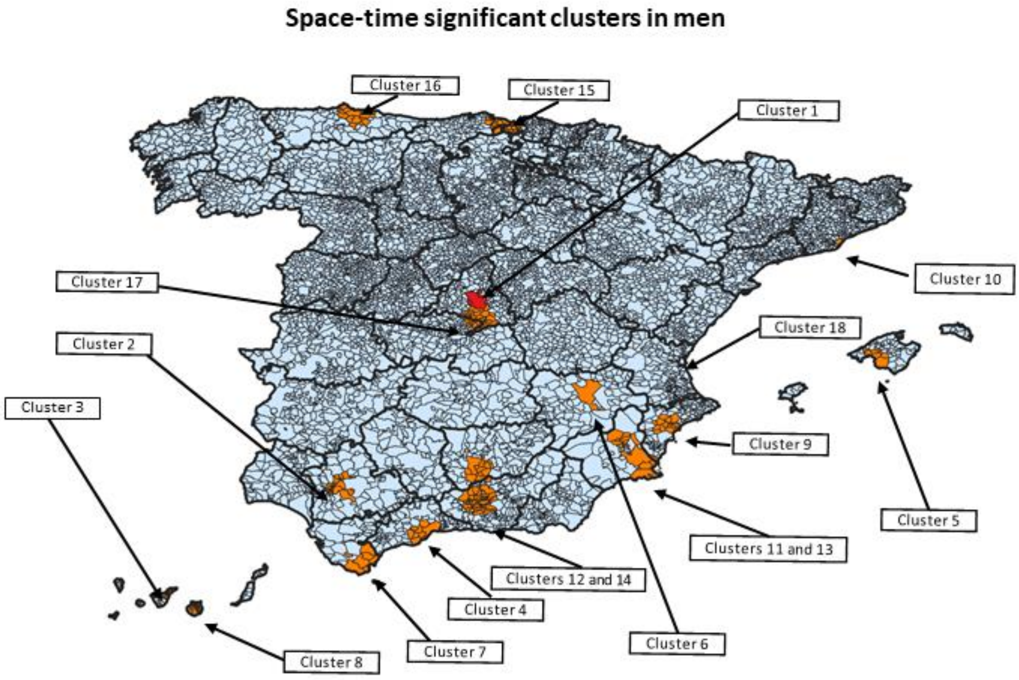

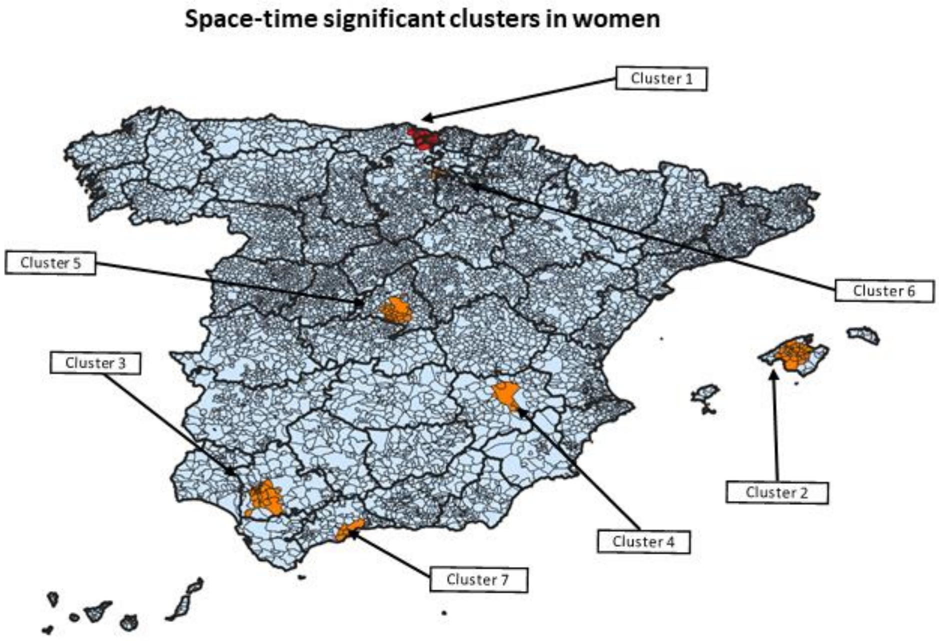

Supplementary Table S1. Space-time significant clusters of hepatitis A in 15-49 years-old men and women, Spain, 2016/2017, maximum temporal cluster size of 40 days

Cluster	Start date	End date	Duration (days)	Observed	Expected	Relative risk	p-value	Total number of municipalities (N classified as Large Urban Area)
**STATISTICALLY SIGNIFICANT SPACE-TIME CLUSTERS IN MEN**								
1	07/02/2017	18/03/2017	39	124	15.449	8.25	< 0.001	3 (3)
2	15/01/2017	23/02/2017	39	68	5.460	12.65	< 0.001	25 (18)
3	15/05/2017	23/06/2017	39	39	3.016	13.05	< 0.001	16 (9)
4	27/02/2017	07/04/2017	39	44	4.952	8.97	< 0.001	13 (8)
5	06/12/2017	06/12/2017	0	12	0.064	187.12	< 0.001	8 (6)
6	07/03/2017	16/03/2017	9	12	0.213	56.39	< 0.001	1 (1)
7	28/04/2017	05/06/2017	38	19	1.343	14.21	< 0.001	8 (4)
8	07/05/2017	15/06/2017	39	29	4.253	6.86	< 0.001	20 (9)
9	17/05/2017	17/05/2017	0	8	0.072	111.07	< 0.001	13 (6)
10	30/05/2017	06/07/2017	37	35	7.266	4.85	< 0.001	1 (1)
11	17/04/2017	25/04/2017	8	8	0.116	69.24	< 0.001	10 (2)
12	29/09/2016	01/11/2016	33	12	0.641	18.78	< 0.001	14 (3)
13	14/06/2017	20/07/2017	36	23	3.719	6.21	< 0.001	8 (3)
14	02/03/2017	10/04/2017	39	20	2.831	7.09	0.0013	51 (31)
15	30/05/2017	06/07/2017	37	18	2.244	8.05	0.0015	41 (17)
16	08/03/2017	12/04/2017	35	17	1.975	8.64	0.0018	13 (9)
17	17/04/2017	04/05/2017	17	19	2.670	7.14	0.0031	31 (14)
18	04/10/2017	04/10/2017	0	3	0.001	2791.78	0.0036	1 (1)
**STATISTICALLY SIGNIFICANT SPACE-TIME CLUSTERS IN WOMEN**								
1	02/06/2017	23/06/2017	21	13	0.10	132.21	< 0.001	31 (7)
2	06/12/2017	20/12/2017	14	13	0.21	63.78	< 0.001	33 (6)
3	24/01/2017	12/02/2017	19	14	0.59	24.17	< 0.001	28 (24)
4	03/03/2017	21/03/2017	18	8	0.07	110.72	< 0.001	4 (1)
5	13/04/2017	18/05/2017	35	26	3.75	7.16	< 0.001	27 (21)
6	22/11/2017	27/12/2017	35	5	0.02	205.91	0.0013	9 (0)
7	28/02/2017	07/04/2017	38	11	0.79	14.06	0.0110	8 (7)

Supplementary Figure S2. Map of municipalities involved in the space-time significant clusters of hepatitis A in 15-49 years-old men and women, Spain, 2016/2017, maximum temporal cluster size of 120 days

Space-time significant clusters in men

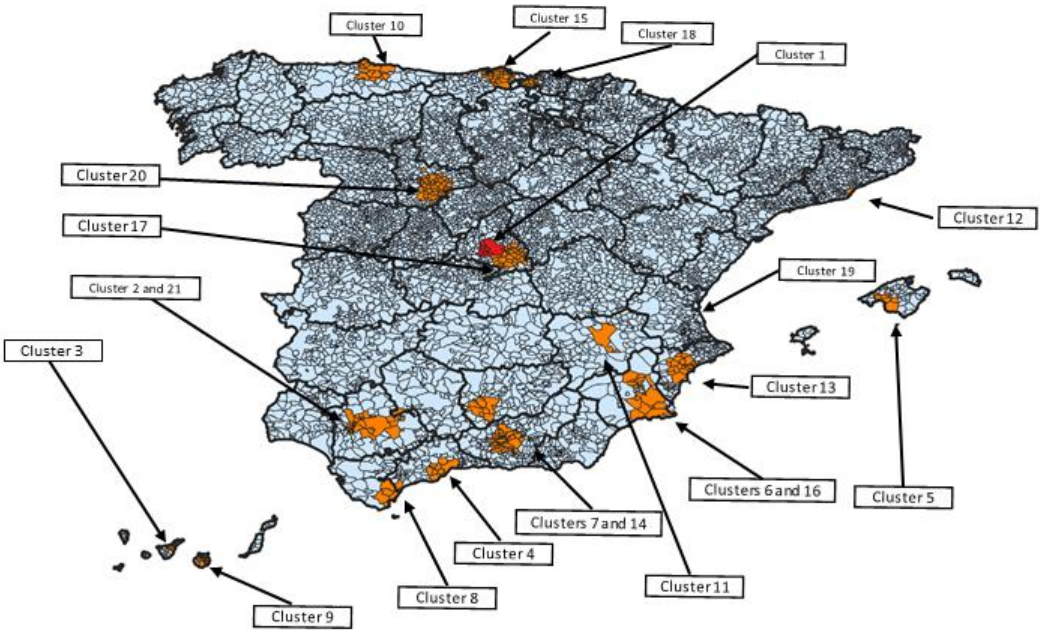

Space-time significant clusters in women

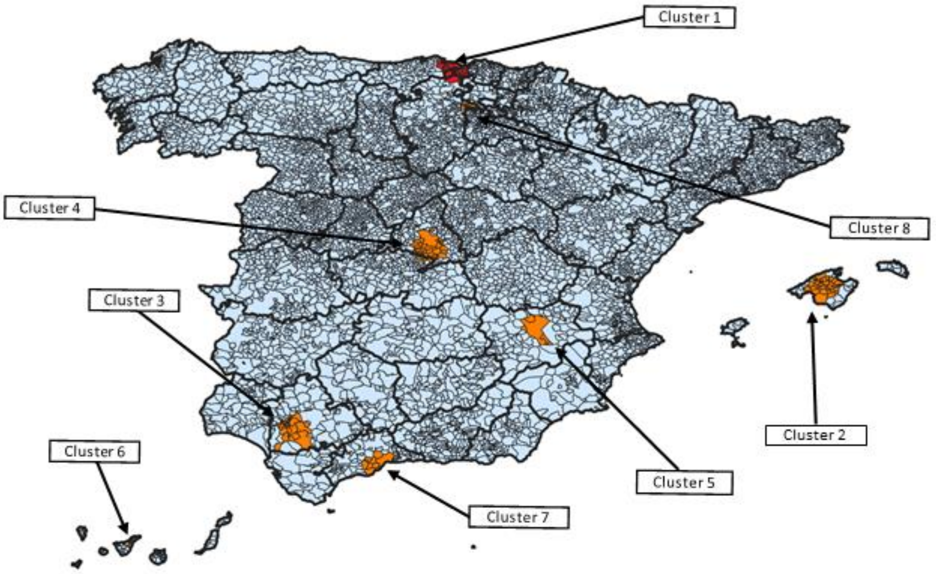

Supplementary Table S2**.** Space-time significant clusters of hepatitis A in 15-49 years-old men and women, Spain, 2016/2017, maximum temporal cluster size of 120 days

**Cluster**	**Start date**	**End date**	**Duration (days)**	**Observed**	**Expected**	**Relative risk**	**p-value**	**Total number of municipalities (N classified as Large Urban Area)**
**STATISTICALLY SIGNIFICANT SPACE-TIME CLUSTERS IN MEN**								
1	23/01/2017	22/05/2017	119	365	56.092	7.06	< 0.001	9 (9)
2	17/11/2016	15/03/2017	118	138	16.987	8.38	< 0.001	28 (19)
3	07/04/2017	04/08/2017	119	91	9.047	10.27	< 0.001	16 (9)
4	06/02/2017	05/06/2017	119	111	14.856	7.66	< 0.001	13 (8)
5	06/12/2017	06/12/2017	0	12	0.064	187.12	< 0.001	8 (6)
6	28/03/2017	24/07/2017	118	56	11.374	4.98	< 0.001	7 (2)
7	01/08/2016	23/11/2016	114	29	2.652	11.01	< 0.001	12 (4)
8	01/02/2017	31/05/2017	119	34	4.045	8.47	< 0.001	9 (4)
9	20/04/2017	17/07/2017	88	45	8.372	5.42	< 0.001	13 (8)
10	15/02/2017	14/06/2017	119	46	9.402	4.94	< 0.001	14 (10)
11	07/03/2017	16/03/2017	9	12	0.213	56.39	< 0.001	1 (1)
12	09/04/2017	18/07/2017	100	67	19.311	3.51	< 0.001	1 (1)
13	22/03/2017	14/06/2017	84	44	9.258	4.79	< 0.001	19 (8)
14	12/01/2017	09/05/2017	117	40	7.783	5.18	< 0.001	47 (27)
15	01/12/2016	28/03/2017	117	28	4.096	6.88	< 0.001	35 (3)
16	17/04/2017	25/04/2017	8	8	0.116	69.24	< 0.001	10 (2)
17	21/03/2017	02/06/2017	73	38	9.839	3.89	0.001	35 (13)
18	20/04/2017	14/07/2017	85	23	3.708	6.23	0.001	20 (12)
19	04/10/2017	04/10/2017	0	3	0.001	2791.78	0.004	1 (1)
20	22/08/2017	07/12/2017	107	25	5.289	4.75	0.016	55 (13)
21	24/10/2016	02/02/2017	101	11	0.826	13.35	0.040	7 (0)
**STATISTICALLY SIGNIFICANT SPACE-TIME CLUSTERS IN WOMEN**								
1	02/06/2017	23/06/2017	21	13	0.10	132.21	< 0.001	31 (7)
2	06/12/2017	20/12/2017	14	13	0.21	63.78	< 0.001	33 (6)
3	24/01/2017	10/03/2017	45	20	1.36	15.13	< 0.001	28 (24)
4	03/03/2017	13/06/2017	102	48	11.26	4.50	< 0.001	38 (25)
5	03/03/2017	21/03/2017	18	8	0.07	110.72	< 0.001	4 (1)
6	16/06/2017	24/09/2017	100	16	1.18	13.90	< 0.001	13 (7)
7	15/12/2016	07/04/2017	113	21	2.52	8.55	< 0.001	13 (8)
8	05/10/2017	27/12/2017	83	6	0.06	106.04	< 0.001	9 (0)
